# Book Reviews

**Published:** 1892-07

**Authors:** 


### Book Reviews.

Annual of the Universal Medical Sciences. A Yearly Report of the Progress of the General Sanitary Sciences throughout the World. Edited by Charles E. Sajous, M. D., and Seventy Associate Editors, assisted by over Two Hundred Corresponding Editors, Collaborators and Correspondents. Illustrated with chromo-lithographs, engravings and maps. Five volumes. The F. A. Davis Company, Publishers, Philadelphia, New York, Chicago, and London. Australian Agency: Melbourne, Victoria. 1892.

This comprehensive and elaborate series of reference books, known as the Annual of the Universal Medical Sciences, appears this year with unusual promptness. The general plan has not been changed from that of the preceding four issues, but it is in every way up to the highest standard of excellence that was fixed at the outset, and each year, the present being no exception, has witnessed an improvement in the details. The associate editors have been carefully chosen with especial reference to their fitness for the work that has been placed upon them, and we find very little to be criticised, either in reference to the general plan of the work or with reference to the methods of its execution. We are somewhat surprised to find that two American publishing firms, owning five or six periodical sheets, have steadily ignored the Annual from the first, refusing even to exchange with it, and prohibiting quotations from the columns of its publications. It is difficult to understand the spirit of anti-progress that pervades some quarters even during the closing years of the nineteenth century, when all scientific minds are saturated with thoughts of harmony and progress and are attempting the highest ideal. However, the Annual can afford to ignore these petty little annoyances, and look trustingly forward to the future that will assuredly give this great work the place which belongs to it amongst the medical literature of the world.

The liberal spirit that controls the editor-in-chief is evidenced by the following extract from his preface:

> No sheet is too unimportant to merit oversight at the hands of the editorial staff. Each periodical, however small, is the representative of a community, of a section, or of a class, and it would certainly be rash to conclude that not a man among them is capable of contributing a point of value worthy of notice.

We have made this quotation to illustrate the fact that no mistake was made when Dr. Sajous projected and put in operation such a comprehensive scheme as the publication each year of a *résumé* of the literature of the world, grouped into volumes, handsomely bound, beautifully printed, and comprehensively indexed. It is a work that must always be found upon the shelves of teachers and authors, as well as men who are in the habit of writing for journals and societies, together with a large mass of the profession who are always interested in keeping abreast of the progress of events. We have spoken in years past of the valuable nature of this work, and we now wish to reiterate all we have said heretofore in its praise, and to add that the present series, the fifth year of its publication, witnesses a triumph in medical literature that would have been thought impossible to attain a few years ago,—one almost amazing to contemplate in its stupendous nature. The publishers and artists have performed their work in a most satisfactory manner, and should not fail to receive due recognition therefor from all who are interested in seeing and owning good literature in handsome covers.

---

REPORT ON CHOLERA IN EUROPE AND INDIA. By EDWARD O. SHAKESPEARE, A. M., M. D., Ph. D., of Philadelphia, United States Commissioner. Transmitted to the Secretary of State. Quarto, pp. xxvi.—945. Washington: Government Printing Office. 1890.

In view of the fact that history teaches us that all previous epidemics of influenza have been followed, in the course of a few years, by an epidemic of cholera, the report of Dr. Shakespeare is most acceptable at this time. It is, by all odds, the most complete work on cholera that has ever been printed in English, and the quality of the work is unsurpassed.

The work is divided into eight chapters, in which are successively treated, The Course of the Last Wide-spread Epidemic of Cholera; The Topography and Demography of British East India in Relation to Cholera; Bacteriological Investigation and Literature; Preventive Inoculation against Cholera; Measures of Prevention, General and Individual; and The Etiology, Pathology, Symptomatology, Prognosis, and Treatment of Cholera, Infectivosa, or Asiatica.

All of these subjects are so well and thoroughly treated that it is impossible to call one more interesting or more valuable than another. The individual taste will be the determining factor here.

Pains should be taken, however, by everyone who reads this book to spread far and wide the opinion of so fair and unprejudiced an observer as Dr. Shakespeare in regard to the preventive inoculation of Dr. Ferrán. This opinion is quite contrary to that expressed by the other foreign commissioners, who seem to have investigated in a most unfair manner, in a spirit antagonistic to Dr. Ferrán, and, therefore, likely to cause a prejudiced and unreliable report.

Dr. Shakespeare says, among other things:

> After having read so much, in the current literature of the day, about the ignorance of Dr. Ferrán of the modern methods of research among bacteria, and of his inability to make decent microscopic preparations, and of his absolute ignorance of the method of staining, I confess that it was with some surprise that I witnessed the facility with which he performed all these operations,—a facility which indicated the *habitual* practice of no mean skill in the performance of these somewhat delicate operations,—and my astonishment was great when, for the first time, I examined with his Hartnack microscope one of the cover-glass preparations which I had seen him make. Honesty and regard for fair dealing require me to say that if there are more beautifully stained microscopic preparations of bacteria, especially of the comma-bacillus of Koch, and of the Finkler and Prior bacillus, anywhere in Europe, I have never seen them. . . . . . Prepared to find the characteristics of a charlatan, I met with nothing in the several interviews which I held with Dr. Ferrán which suggested anything of the kind. I found him to be a quiet, reserved, courteous, intelligent, and generally well-informed physician. The impressions which I formed of his theoretical knowledge of bacteria, and of the modern methods of research in the field of natural history, have compared very favorably with those of most of the leading bacteriologists with whom I have come in contact.

After reviewing carefully the official reports, he concludes:

It would seem, therefore, from careful analysis of the official statistics, that the practice of the anti-choleraic inoculation, after the method of Ferrán, besides giving the subject inoculated a considerable immunity from attack and death by cholera, furnishes a means of bringing an epidemic rapidly to an end.

In the chapter on Measures of Prevention, General and Individual, a most thorough review of the subject, from all points, is given, and practical measures for national quarantine and individual prophylaxis are presented.

In the last chapter, the section on Treatment naturally attracts us most. It begins with the statement that

Knowledge of efficient methods of treatment of cholera infectivosa has by no means kept pace with that of the etiology and prophylaxis of the disease. Unless the so-called methods of hypodermoclysis and enteroclysis shall prove as effective as the recent experience of some Italian observers would seem to indicate, there appears to have been no marked advance made in the therapeutics of severe attacks of cholera. . . . . . Whilst this is true of severe attacks of cholera, nevertheless there is scarcely any grave disease which is more manageable, if it be properly and promptly treated during the earlier stages.

The best methods of treatment are then reviewed, and two full pages are given to descriptions of the methods of treatment known as enteroclysis and hypodermoclysis. These methods seem rational, and are certainly worthy of trial. Cantani, the Italian physician, who introduced these methods of treatment, says:

The physician who knows how to use, with courage and reliance, laudanum, tannic enteroclysis, warm baths, and hypodermoclysis, will have to record among the victims of cholera only those unfortunates who, when he was called, were already well advanced in the stage of cyanosis and collapse.

A number of maps, diagrams, and charts, illustrating various matters treated of in the text, add greatly to the value of the work, which is made in every respect convenient for reference by the addition of a most exhaustive index. Dr. Shakespeare is deserving of the highest praise for the thoroughness of his work; the medical profession is to be congratulated that so able and worthy a representative was chosen for this important task, and President Cleveland is to be thanked for the excellent choice that he made.

DeL. R.

THE PRINCIPLES AND PRACTICE OF MEDICINE. Designed for the Use of Practitioners and Students of Medicine. By WILLIAM OSLER, M. D., Fellow of the Royal College of Physicians, London, Professor of Medicine in the Johns Hopkins University, and Physician-in-Chief of the Johns Hopkins Hospital, Baltimore. Formerly Professor of the Institutes of Medicine, McGill University, Montreal; and Professor of Clinic Medicine in the University of Pennsylvania, Philadelphia. New York: D. Appleton & Co. 1892.

Departing from the usual custom of dividing his book into chapters, Osler, without introduction and with only a small prefatory note, commences at once with his subject, and introduces us to his work, made up of eleven sections, by first considering specific infectious diseases. These are considered in thirty subsections, occupying 269 pages. In view of the recent investigations concerning tuberculosis, it is probable that what Osler has to say on this subject will attract much attention. Singularly enough, however, he dismisses the investigations of Koch in a very short paragraph, during the course of which he says, "It will probably be several years before we can speak with decision upon the true position of this remedy," but quotes Schede as saying that it has very positive value in tuberculous arthritis. Osler is very direct and positive in his assertion that the outlook in tuberculosis depends much upon the digestion. This disease has so long been considered from the standpoint of malnutrition, and has been recorded as a malady of decided tendencies to retrograde tissue metamorphosis, that we have come to look upon the care of the digestive tract as one of the principal avenues to improvement, especially in the early stages of this disease. Given a digestive tract that refuses to assimilate hydro-carbons, and it is almost impossible to obtain benefit from any kind of treatment. We are in accord with Osler in his estimate of arsenic as one of the most satisfactory general tonics that can be administered in tubercular disease.

In section two we find constitutional diseases considered in twelve sub-sections. We are much interested in what this author has to say on the subject of rheumatism, because we find so many sufferers from this disease, either real or imaginary, true or false, —acute, chronic, articular. We must confess that we have not been able to draw any new inspiration from Osler's handling of the subject. It is a popular notion among the profession that cardiac complications are very apt to follow in the trail of acute rheumatism. One would suppose, from the shallow treatment of the subject by Osler, that this was of very little consequence as a possible complication, not often to follow and still less often to be of dangerous portent. One of the most interesting chapters in the practice of medicine that we have lately seen is the section here devoted to diseases of the digestive system, under ten sub-divisions, where one will find the several diseases of the mucous, serous, and glandular system most ably considered. Diseases of the tonsils are always full of professional interest, since these cases are so frequent, manifested either in an acute or chronic form, and a physician will often build his reputation on the prompt relief of the one variety or the other of this distressful malady.

We are finding ourselves more and more in accord with Osler when he says, "If the tonsils are large, and the general state is ever influenced by them, they should be at once removed.'' In our early professional life we came to this opinion, but later the more conservative view was unfolded to our vision, and we thought the operation of tonsillar excision almost absolutely unnecessary; now, however, after more mature observation and experience, we are inclined to coincide with Osler's opinion in the sentence just quoted.

Diseases of the stomach are justly demanding and obtaining more consideration, more study, more analysis, and more satisfactory handling than ever before. Osler handles these diseases as satisfactory, perhaps, as possible in the limits that he allots to their consideration, and he is especially pointed in some of his asseverations, but we are unable to gain therefrom much knowledge as to the management of so-called dyspepsia, which may be either new or striking in its character. Osler follows Leube with considerable closeness in reference to the ingestion of the foods that are permissible in the several forms of stomach disease.

The diseases of the respiratory system are next considered in five sub-sections, and this is, perhaps, as attractive as any part of Osler's book. We cannot, in the space that we find ourselves necessarily limited to, give an analysis of the several diseases treated of, but we have read carefully what Osler has to say relating to nasal and autumnal catarrh, hoping that we might receive some hint that would enable us to estimate as to the value of the writings of rhinologists on this subject. Osler gives credit to Roe, of Rochester, *inter alia*, for his investigations on the subjects of hay fever, and particularly does he credit Daly, of Pittsburgh, with the discovery of the fact that, in a large proportion of the cases of hay asthma, there is local disease of the mucous membrane of the nose. There can be no doubt that numerous asthmatic and other deep-chest symptoms are in reality reflexes of nasal or pharyngeal disease, and we wish that Osler had given us some new light on this point, which, in our opinion, is one of the most interesting branches of the subject.

Pleurisy is interestingly handled by Osler, although, in view of the fact that this is an exceedingly common disease, and especially are its sequelæ frequently overlooked, we believe that more complete justice might have been done the subject.

Section five considers diseases of the circulatory system, and the two branches thereof of chief importance, namely, endocarditis and valvular diseases, are handled with much address and great satisfaction. We commend these two sub-sections to every teacher of internal medicine, and especially to the expert in diseases of the chest.

The diseases of the kidneys are treated of in section seven, and here again, on some points, Osler is especially direct and satisfactory. The practitioner will find much here to read with care and to ponder over.

Section eight is devoted to the discussion of nervous diseases, section nine to diseases of the muscles, and section ten to the intoxications. Under section eight are included, (1) diseases of the nerves, (2) diseases of the spinal cord, (3) diseases of the brain, (4) general and functional diseases, and (5) vaso-motor and trophic disorders—or, in other words, diseases of the nervous system proper. Generally, authors have included the muscular diseases and the intoxications under nervous diseases, but Osler very wisely separates them. Although the discussion of these subjects must be brief, they are, nevertheless, very compact and comprehensive. Osler's classification and order is excellent, and therein lies the value of a text-book.

Under papillitis the author says it is present in over ninety per cent. of intracranial tumors, and that it is not due to pressure, as many suppose, but to a descending neuritis. The treatment of the various affections is honest and conservative, considerable importance being laid upon the rest treatment. The quotations of many American authors forms a pleasing part of these sections.

The remaining sections of this work — IX., X., XI.,— deal with diseases of the muscles, intoxication, sunstroke, obesity, and diseases due to animal parasites, respectively. A comprehensive index of twenty-eight pages closes the volume. It is one of the most important contributions to the literature of the principles and practice of medicine that has appeared from an American author for many years, and will, undoubtedly, assume a permanent place on the list of college text-books in the very near future. The publishers have executed their work in excellent taste, and are to be commended for their enterprise in putting it forth at a time when so much literature is being sent out and the risks of text-book publication are very great.

---

History of the Circumcision from the Earliest Time to the Present. Moral and Physical Reasons for its Performance, with a History of Eunuchism, Hermaphrodism, etc., and of the Different Operations Practised upon the Prepuce. By P. C. Remondino, M. D. (Jefferson), Member of the American Medical Association; of the American Public Health Association; of the San Diego County Medical Society; of the State Board of Health of California, and of the Board of Health of the City of San Diego; Vice-President of the California Medical Society, and of the Southern California Medical Society, etc. Number 11 in the Physicians' and Students' Ready Reference Series. Small 8vo, pp. 346. Philadelphia and London: F. A. Davis. 1891.

The value of this book as an addition to our medical literature must largely depend on the spirit in which it is received by the profession. The leading idea of the hygienic effect of circumcision, under certain circumstances, has been recognized in all ages, and there has not been wanting those even among the uncircumcised who have seen in its wise provisions, under climatic and other conditions obtaining in the Orient, the hand of Providence himself. Moses certainly was a wise legislator, by whatever influence guided, and his rules for the physical well-being of his charge leave little room for improvement, even in our own scientific age. This may be conceded, however, without admitting the full claim of the learned writer as to the propriety of circumcision under all ordinary circumstances, and that its omission is attended with danger and even dire results. Remondino is apparently an enthusiast in favor of his theory, and in his eagerness to establish his point scarcely hesitates to refer nearly all the ills of man's moral and physical nature to neglect in adopting his favorite remedy. We think he claims too much of immunity from disease for those who practise circumcision as a rite; it may be true that the Jewish race are physically stronger than those who are called Christians, and it may even be true that circumcision is a factor in their favor, but if it be, we think it is too small a one to count for much among the many others that might be found by careful and unbiased investigation. The prepuce is particularly blamed for a large class of nervous disorders, and numerous cases are cited which seem to indicate that this is not without foundation, but the propriety of a surgical operation as a prophylaxis, in the absence of any indication or symptom, would seem, from a surgical point of view, to be an officious proffer of assistance to Nature when she has not called for help. Notwithstanding these objections, the book must be commended for the exhaustive treatment of the subject. The many important hints and suggestions which it contains, and the history of the various modes of operation practised from the remotest historic times to those in vogue among the most learned surgeons of our time, make it a valuable addition to the library. Copious notes and a good index finish the volume.

---

TRANSACTIONS OF THE AMERICAN SURGICAL ASSOCIATION. Volume the IX. Edited by J. EWING MEARS, M. D., Recorder of the Association. Pp. xxxii.—508. Philadelphia: Printed for the Association, and for sale by William J. Dornan. 1891.

This volume is the record of the work of this celebrated association done in Washington at the second triennial Congress of the American Physicians and Surgeons. It was to be expected that under the guidance of its distinguished president, Dr. Claudius H. Mastin, of Mobile, the scientific excellence of this volume would be at least equal to any which the society has issued. Whoever will carefully examine its pages will easily come to the conclusion that this high expectation has been more than realized. The papers, for the most part, have been published in abstract in the journals, and, consequently, need not be dealt with *seriatim* in this notice. We cannot, however, let the opportunity pass to commend the most excellent address of the president, which we listened to with profound interest, and whose eloquent words are still ringing in our ears. His recommendation that a statue be erected to the memory of the late Dr. Samuel D. Gross, has taken root and assumed a fixed and determined form. A circular has been issued to the profession, and the proper committees have been appointed to put the matter in the way of accomplishment.

The first scientific paper in the book is by the late Dr. D. Hayes Agnew, on the Present Status of Brain Surgery, and is a scholarly and able analysis of the work done in that special line by the surgeons of Philadelphia. Dr. Nicholas Senn presented the subject of Treatment of Tuberculosis of Bones and Joints by Parenchymatous and Intra-Articular Injections. This, and the paper by Dr. White on Surgery of the Spine, attracted much attention, and led to valuable discussion. Dr. Albert Vander Veer, of Albany, presented an exhaustive paper on Retro-Peritoneal Tumors, that deserves to be carefully read by every abdominal surgeon. The final paper of the volume, by Dr. George Ryerson Fowler, on Aseptic Operative Technique, is a careful *résumé* of this subject, and contains many points of interest, because of their originality. We close by saying, as we began, that this is one of the most excellent of the many good volumes that this association has issued.

---

PYE'S SURGICAL HANDICRAFT. A Manual of Surgical Manipulations, Minor Surgery, and other matters connected with the work of House-Surgeons and Surgical Dressers. With 300 illustrations on wood. First American, from the third London edition. Revised and edited by T. H. R. CROWLE, F. R. C. S., Surgical Register to St. Mary's Hospital; and Surgical Tutor and Joint Lecturer on Practical Surgery in the Medical School. Complete in one volume. New York: E. B. Treat, 5 Cooper Union. 1892.

This work is intended primarily for house-surgeons and dressers, and it is for this very reason that it is a valuable addition to the surgical library. It takes up so many little points that the more pretentious works on surgery pass over and leave to the understanding of the reader, that it must prove of great benefit to those practitioners who have not had the advantage of hospital training, and to whom, therefore, the larger books do not convey the definite and precise information so essential in obtaining good results. The author takes up hemorrhage, to which he devotes several chapters. The especial kinds of hemorrhage and the treatment of each are accurately and fully described, and form one of the most valuable sections of the book. The sections also upon bandages, splints, etc., and fractures, are explicit, and their value much increased by numerous illustrations. Sections on wounds, ulcers, burns, etc.; on cases requiring prolonged or mechanical treatment; on surgical and general emergencies; on anesthetics; on extraction of teeth and ear diseases; on minor surgery and kindred subjects, and on preparation of patients for operation and their after-treatment, making of poultices, fomentations, etc., and surgical case-taking, with an appendix containing an excellent formulary, complete the volume and form a collection of topics of special value to the medical practitioner. The wood-cuts, some 300 in number, are of standard excellence; the type is clear, and the general arrangement of the book good. The marginal notes are very convenient and save much time in getting at the contents of a page.

J. P.

---

Lectures on Tumors from a Clinical Standpoint. By John B. Hamilton, M. D., LL.D., Professor of Principles of Surgery and Clinical Surgery, Rush Medical College, Chicago ; Professor of Surgery, Chicago Polyclinic ; Surgeon, formerly Supervising Surgeon-General, United States Marine Hospital Service ; Surgeon to Presbyterian Hospital, Chicago; formerly Professor of Surgery in Georgetown University ; Surgeon to Providence Hospital, etc., etc. For the use of the Students. Second edition. The Physicians' Leisure Library. Price, cloth, 50 cents ; paper, 25 cents. Detroit, Mich.: Geo. S. Davis. 1892.

The appearance of the second edition of this valuable little book within a twelvemonth from the first issue, attests the value and usefulness of the volume in a manner far more substantial than a mere description of the book. The author has taken occasion to correct some of the typographical faults of the first edition, and also to eliminate some of the incongruities that were due to imperfect proof-reading and hasty preparation. We expressed our opinion of this book in unmistakable terms in No. 4, Vol. XXXI., of the Journal, and see no reason to change our opinion after a more mature consideration of the subject and a further examination of this second edition. We believe that it will be accepted by the profession as a valuable addition to the literature of the subject on which it treats, and shall not be surprised to find further editions called for in a reasonable time.

---

Transactions of the American Association of Obstetricians and Gynecologists. Volume IV. For the year 1891. Standard octavo, pp. xlvi.—320. Philadelphia: William J. Dornan, Printer. 1892.

This handsome volume comprises the proceedings of this prominent Association, held at the Academy of Medicine in New York, September, 1891. The papers, for the most part, are of a superior order, and the discussions upon them are exceedingly strong. It is a kind of work that must give stimulus to medical societies all over the land, and should be read by every physician interested in the progress of obstetrics, gynecology, and abdominal surgery, no matter whether he poses in the attitude of a specialist or not. The volume before us is handsomely illustrated with numerous wood-cuts and process plates, which give an artistic touch to the subjects that they elaborate, and facilitate a clear understanding of the text. The next meeting of this Association is announced to be held in St. Louis, September 20, 21, and 22, 1892, and we may confidently expect another admirable volume as the result of that meeting.

---

TRANSACTIONS OF THE NATIONAL ASSOCIATION OF THE RAILWAY SURGEONS. Held at Buffalo, N. Y., April 30 and May 1, 1891. With a Historical Sketch of the Association, List of Members, and other Matters Relating to the Association. Illustrated with portraits of the officers of the association and others. Published by the *Railway Age and Northwestern Railroader*, Chicago, Ill.

This volume is a record of the proceedings of this association at its fourth annual meeting, held in Buffalo, April 30 and May 1, 1891. The scientific papers read at the meeting, have heretofore been published either in abstract or as a whole in the several journals, so that the profession has already obtained a knowledge of the work done at the meeting and the value thereof. The growth of this association has been somewhat phenomenal, comprising now about 1,200 ; whereas, at the Buffalo meeting the membership numbered in the vicinity of 300. This book is handsomely printed, and contains many portraits of the members of the association, but we may express the surprise where we see so much material to draw from, that there should not be more scientific value to the illustrations and less of the personal element.

